# Does Cosleeping Contribute to Lower Testosterone Levels in Fathers? Evidence from the Philippines

**DOI:** 10.1371/journal.pone.0041559

**Published:** 2012-09-05

**Authors:** Lee T. Gettler, James J. McKenna, Thomas W. McDade, Sonny S. Agustin, Christopher W. Kuzawa

**Affiliations:** 1 Department of Anthropology, University of Notre Dame, Notre Dame, Indiana, United States of America; 2 Department of Anthropology, Northwestern University, Evanston, Illinois, United States of America; 3 Cells to Society (C2S): The Center on Social Disparities and Health, Institute for Policy Research, Northwestern University, Evanston, Illinois, United States of America; 4 USC-Office of Population Studies Foundation, University of San Carlos, Cebu City, Philippines; University of Washington, United States of America

## Abstract

Because cross-species evidence suggests that high testosterone (T) may interfere with paternal investment, the relationships between men's transition to parenting and changes in their T are of growing interest. Studies of human males suggest that fathers who provide childcare often have lower T than uninvolved fathers, but no studies to date have evaluated how nighttime sleep proximity between fathers and their offspring may affect T. Using data collected in 2005 and 2009 from a sample of men (n = 362; age 26.0 ± 0.3 years in 2009) residing in metropolitan Cebu, Philippines, we evaluated fathers' T based on whether they slept on the same surface as their children (same surface cosleepers), slept on a different surface but in the same room (roomsharers), or slept separately from their children (solitary sleepers). A large majority (92%) of fathers in this sample reported practicing same surface cosleeping. Compared to fathers who slept solitarily, same surface cosleeping fathers had significantly lower evening (PM) T and also showed a greater diurnal decline in T from waking to evening (both p<0.05). Among men who were not fathers at baseline (2005), fathers who were cosleepers at follow-up (2009) experienced a significantly greater longitudinal decline in PM T over the 4.5-year study period (p<0.01) compared to solitary sleeping fathers. Among these same men, baseline T did not predict fathers' sleeping arrangements at follow-up (p>0.2). These results are consistent with previous findings indicating that daytime father-child interaction contributes to lower T among fathers. Our findings specifically suggest that close sleep proximity between fathers and their offspring results in greater longitudinal decreases in T as men transition to fatherhood and lower PM T overall compared to solitary sleeping fathers.

## Introduction

Humans are one of the few mammalian species in which fathers are heavily invested in their offspring and, specifically, often assist mothers in the direct care of young [1]. Like other mammals in which fathers care for offspring, human males must shift behavioral and energetic priorities after becoming parents in order to fulfill the requirements of paternal investment [2], [3]. In particular, because time and energy are limited commodities [4], males in these species must navigate trade-offs between conflicting behaviors related to mating effort (e.g. competing with conspecific males, attracting females, guarding mating partners) and parental investment (e.g. provisioning, feeding, grooming, protecting offspring) [3]. Based on cross-species data, it is widely assumed that the hormone testosterone (T) plays a primary role in mediating such shifts in reproductive strategy between mating and parenting. In particular, because T has been found to facilitate and enhance male mating effort through its influences on traits such as skeletal muscle and ornamentation as well as behaviors related to competition with other males and attraction of females [5]–[9], high T may conflict with effective fathering [10]–[13], potentially reducing offspring well-being and survival.

There is growing evidence that this model may apply to human males. For example, men with elevated T show greater skeletal muscle mass [14], particularly if they are physically active [15], which may reflect energetic investments in mating effort [5], [16]. Higher T has been shown to moderately relate to aggressive behaviors and personality characteristics that may enhance pursuit of social dominance [6] and has also been linked to motivation to win in competitive events [17], [18], extraversion [19], and sensation seeking [20], [21]. Moreover, elevated T predicts heightened risk-taking [22], [23] and greater likelihood to engage in health-compromising display behaviors [24] as well as drug and alcohol abuse [25]. Men with higher T have also been found to have a greater number of lifetime sexual partners [26]. In addition, in an experimental study, men with greater T reported lower sympathy or need to respond to infant cries relative to men with lower T [27], and, in a separate, similar study, men's T decreased in conjunction with providing a nurturing response to infant cries [28]. Recently, low sensation seeking fathers were also shown to have reduced T relative to high sensation seeking fathers [29]. Fathers with lower baseline T have also been found to engage in more hands-on caregiving behaviors in observed parent-child interactions [30]. Collectively these findings suggest that elevated T likely facilitates somatic growth and behavioral priorities that enhance men's mating effort but may also reduce resources fathers have available for parental investment and diminish their sensitivity to offspring needs.

Although in some cultural settings there appears to be little to no relationship between fatherhood and T [13], [31] or lower T appears more strongly correlated to being married rather than being a parent [32], [33], multiple cross-sectional studies have found that fathers have lower T than non-fathers [29], [34]–[36], and there is increasing evidence that differences in T between fathers and non-fathers are greater in cultural settings in which fathers participate in direct care of their children [13], [37]. Moreover, using data from the same study from which the present sample was drawn, we also recently showed that the transition from being single and childless to being a partnered father caused T to decline longitudinally in a sample of men in the Philippines, with fathers who reported providing no childcare having higher T than fathers who participated extensively in caregiving [38]. Taken together these results suggest that direct interaction with children may be an important cause of the lower T often observed among fathers.

One important way that fathers may interact with their offspring is through cosleeping [12], [39], which involves close proximity with their offspring during sleep. To date, little work has evaluated the effects of cosleeping on human male physiology or behavior. Specifically, cosleeping is an umbrella term that defines any sleeping arrangement in which a child and his/her caregiver sleep in close enough proximity to engage in and communicate through sensory exchanges [40]. Although expression varies across cultures, two fundamental forms of cosleeping are “roomsharing,” in which a child sleeps near his/her caregiver(s) but on a separate surface, and “same surface cosleeping,” in which a child shares a sleeping surface with the caregiver(s) [41]. Because much past research on cosleeping has been conducted in the US, Australia, New Zealand, and Europe, same surface cosleeping is also often simplified to “bedsharing” in the scholarly literature on the subject, though in many cultures in which it is common families do not sleep on adult beds involving a thick mattress and box spring [42], [43]. Barry and Paxson's report on cross-cultural behavioral patterns, spanning 127 cultural groups, revealed that mothers and children sleep in the same room in 79% of societies and mothers specifically shared a sleeping surface with their children in 44% [43], [44]. However, much less is generally known about the role and placement of the father in cosleeping arrangements cross-culturally [45]. In one of the only large surveys to have examined fathers' placement during nighttime sleep, it was shown that when mothers sleep with their infants, a majority of the time fathers are also present [46], although this study was conducted in the US, where routine cosleeping is not necessarily the norm. Though available data are limited, preliminary observations also suggest that this pattern of fathers being present with mothers and children during sleep may also be common in cultures where cosleeping is more widely practiced [47]–[49].

Research on the physiological and behavioral implications of familial sleeping arrangements has focused almost entirely on mother-infant cosleeping, particularly as it contributes to promotion of breastfeeding. Although variation in sleep quality can influence men's health [50] and physiology [51] as well as mental well-being for fathers of young children [52], comparatively little is known about the impacts of different familial sleep arrangements on paternal behavior and biology. In two existing studies of familial sleep behavior, bedsharing fathers showed less synchrony with their infants' arousals, compared to mothers, and generally slept beyond arm's reach of their infants [45], [53]. These studies took place in Britain and New Zealand with samples drawn from populations among which same surface cosleeping was not necessarily the predominant sleeping practice, and it is unclear whether their findings extrapolate to societies in which bedsharing is more culturally normative and fathers may sleep closer to their children and be more routinely involved with nighttime care. Daytime father-child contact and proximity have been found to predict lower baseline T among fathers living in several cultural settings [13], [37], [38]. However, notably, short-term (within 20–30 min) father-child interactions have not been observed to cause acute declines in paternal T [30], [54], including in a sub-set of fathers from the sample we analyze here [55]. Thus, the mechanisms or pathways linking fathers' T and their childcare behaviors remain somewhat unresolved. To our knowledge, no prior work has investigated whether T may differ between fathers based on whether they sleep separately from their children or whether they sleep in close proximity to them.

To test this hypothesis, we drew on data from a large study conducted in the Philippines, where same surface cosleeping, at least between mothers and infants, appears to be a common practice [44], [56]–[58]. Specifically, we analyzed data collected in 2005 and 2009 from fathers (n = 362) in the Cebu Longitudinal Health and Nutrition Survey, a population-based birth cohort study that began in 1983–1984 in Cebu City, Philippines. Here, using data collected in 2009, we test whether fathers who sleep next to their children (same surface cosleeping) have lower T compared to men who sleep in a separate room from their children. Focusing on men who were non-fathers at baseline (2005), we also test whether baseline T predicts which men will become solitary sleeping or cosleeping fathers by follow-up (2009) and how familial sleep practices predict change in T between baseline (2005) and follow-up. Finally, because previous analyses from this cohort have suggested that fathers of younger offspring have lower T [38], we also consider whether the nature of relationships between offspring age and paternal T vary based on familial sleeping patterns.

## Materials and Methods

### Study population

Data were collected in 2005 and 2009 as part of the Cebu Longitudinal Health and Nutrition Survey (CLHNS), a population-based cohort study of mothers and their infants born in 1983–84 in Metro Cebu, which encompasses the urban center of Cebu City and other large adjacent cities as well as rural, mountainous areas. The original 1983–84 survey was conducted in 17 urban and 16 rural barangays (neighborhoods) [59]. As of 2009, 68% of the subjects in the present study reported living in urban barangays. The male cohort participants were a mean of 21.5 ± 0.3 (SD) and 26.0 ± 0.3 years old at the time of data collection in 2005 and 2009, respectively. Socio-economic and behavioral data were collected using questionnaire-based, in-home interviews in the local dialect [59]. Because having adopted or step-children is rare for Cebuano men in their twenties, men were defined as fathers if they reported having one or more biological children [38]. This selection criterion eliminated 5 subjects who only had adopted or step-children. All 5 of these subjects reported cosleeping with their non-biological children. Men whose youngest child was 1 year old or less were defined as fathers of infants. Self-reported psychosocial stress in the month preceding sampling was quantified via a modified version of the 10-item Perceived Stress Scale (PSS) [60]. Men indicated their recent (2007–2009) illness history by reporting whether they had ever been sick since the last CLHNS survey (2007) and whether they had been hospitalized during that period. Triceps skinfold thickness (mm) were measured using standard anthropometric techniques [61]. Fat-free mass (kg) was calculated after body fat percentage was derived using triceps skinfold thicknesses, body density estimates, and a body composition predictive equation [62]. Men self-reported their sleep location relative to their offspring on the night before the interview and also reported their usual wake and bed times. Using these reports, men were stratified into 3 familial sleeping arrangements: those who reported sleeping in a separate room from their children (solitary sleepers), those who slept in the same room but not on the same surface (roomsharers), those who shared a sleeping surface with their child (same surface cosleepers).

### Ethics statement

This research was conducted under conditions of informed consent with human subjects clearance from the Institutional Review Boards of the University of North Carolina, Chapel Hill and Northwestern University. Written informed consent was received from all participants.

### Sample selection

During the 2009 survey, 908 males of the original 1983–84 cohort of 1633 liveborn males were located and interviewed. Because the present analysis focuses on familial sleep arrangements, the sample was initially limited to the 446 men who identified themselves as fathers. Men who had sleeping patterns consistent with shift work or who had spent less than 8 hours or greater than 20 hours awake on the day of sampling, which may increase the likelihood of disrupted circadian rhythms for T [51], [63], were eliminated from the sample (n = 21). Fathers were excluded if they reported having no contact or not residing with their child(ren) (n = 51). One subject was excluded because of a T value below the assay detection limit while a second subject was eliminated because of T value 9 SD above the sample mean. A final sample of 362 men had all required data and met the criteria for the present analysis. Men in this sub-sample were born to slightly less educated mothers (average grade completed: 7^th^ grade vs. 8^th^ grade; p<0.001) compared to other CLHNS male subjects, but did not differ from excluded individuals on household income, household size, birth order, mother's height, or birth weight and length (all p>0.1).

### Paternal caregiving

The 19 paternal caregiving behaviors about which fathers were asked were drawn from a previous large-scale survey on male parenting behaviors in the Philippines. Examples of the caregiving behaviors included: feeding children, playing, bathing children, reading to children, and walking children to school. Men estimated how much time they had spent on each activity in the last 7 days, and the total was divided by 7 to create a variable for caregiving per day [55].

### Saliva sample collection

At both time points, on the day of their in-home interview, participants were provided with instructions and two polypropylene tubes for saliva collection. Subjects were asked to refrain from brushing their teeth, eating, drinking alcoholic, caffeinated, or other non-water beverages, exercising, taking medication, and smoking in the 30 minutes prior to sampling [55]. The subjects were not instructed to rinse their mouths prior to sampling. The first sample was collected immediately prior to bed (PM) on the interview day. After collection, they sealed the tube and kept it at room temperature. Mean PM sampling time was 9:19 PM ± 3:30 (SD) in 2005 and 9:43 PM ± 1:23 (SD) in 2009. They were instructed to place the second tube next to their bed and to collect the second sample immediately upon waking the following morning (AM). Thus, the evening sample was collected the night before the morning sample, which allowed us to schedule single interviewer follow-up visits to each participant to collect both samples. Respondents reported time of saliva collection, wake time on the day of sampling (2005), and usual wake time. Our subjects conformed closely to the protocol, as their self-reported waking time (2005) was 6:42 ±1:56 and their AM sampling time (2005) was 6:43 AM ± 1:56. Mean AM sampling time was 6:32 AM ± 1:24 in 2009. Saliva tubes were collected later the second day by an interviewer, who placed the tubes on ice packs in a cooler while in transit to freezer storage at −35 C. They were shipped on dry ice to Northwestern University, where they were stored at −80 C.

### Salivary T assessment

T concentrations were determined at the Laboratory for Human Biology Research at Northwestern University using an enzyme immunoassay protocol developed for use with saliva samples (Salimetrics, State College, PA; Kit No. 1-2402). Interassay coefficients of variation were 13.7% and 11.5% for high (200 pg/mL) and low (20 pg/mL) kit-based control samples, respectively, in 2005 samples and 7.8% and 17.9% for high and low control samples, respectively, in 2009 samples.

### Statistical analyses

All analyses were conducted using version 10 of Stata (Stata Corporation, College Station, TX). AM T (pg/mL), PM T (pg/mL), diurnal and longitudinal change in T (pg/mL), duration of marriage and fatherhood, number of children, household size, educational achievement (highest grade completed), sleep duration, anthropometric measures, and PSS were all analyzed as continuous variables. Prior to the calculation of absolute change in T between baseline (2005) and follow-up (2009), baseline AM and PM T measures were adjusted for marital status and time of sampling. These adjustments were conducted by separately regressing AM and PM T on time of sample collection and marital status, predicting the model's residuals, and adding the original dependent variable's (e.g. AM T) mean to the residuals, which removes the effect of the independent variable on the dependent variable. Similarly, 2009 AM and PM T were adjusted for time of saliva collection using this technique prior to statistical modeling. Diurnal change in T was calculated as (PM T minus AM T). As noted above, our PM sample was collected before nighttime sleep on the day of the interview, with the AM sample being provided the following morning. As a consequence the relationship between the AM and PM samples used to calculate diurnal change in T differs from other studies in which the AM (first sample) and PM (second sample) collections occur on the same day [e.g. 64].

We first compared fathers, stratified according to their familial sleeping arrangements, on a series of socio-economic, demographic, and behavioral variables using ANOVA or Fisher's exact test (Table 1). For variables that were strongly rightly skewed, we used either Poisson or negative binomial regression to assess group differences. We then applied multiple linear regression to predict AM and PM T as well as the diurnal change in T from familial sleeping arrangements, controlling for covariates and confounders, including self-reported psychosocial stress, nighttime sleep duration, and self-reported sleep quality. Focusing on men who were non-fathers at baseline (2005; n = 209), we then used logistic regression to assess whether men's baseline T predicted whether they slept solitarily or coslept at follow-up in 2009. Drawing on this same sub-sample (n = 209), we also used multiple linear regression to test whether familial sleeping arrangements predicted longitudinal change in T between baseline and follow-up. Finally, applying one-way ANOVA with Bonferroni *post-hoc* multiple comparison tests, we assessed whether having an infant-aged offspring influenced T based on familial sleeping arrangement.

**Table 1:** Descriptive statistics.

	Familial nighttime sleep practices			
	Solitary sleeping	Roomsharing	Same surface cosleeping	
	(n = 17)	(n = 11)	(n = 334)	p value^a^
**Socio-economic variables**				
Educational attainment^b^	10.6±3.4	12.1±4.6	11.2±5.1	0.74
Presently employed (%)	71%	91%	80%	0.47
Number of people in household^c^	4.5±1.7	4.5±1.8	5.5±2.7	0.09
Urban resident (%)	65%	91%	68%	0.27
**Marital and fatherhood variables**				
Married/cohabitating (%)	94%	100%	99%	0.22
Duration of relationship (years)	3.8±2.6	5.1±2.6	3.9±2.2	0.19
Time as a father (years)^c^	3.3±2.5	4.7±2.6	3.3±2.2	0.10
Weekly sexual intercourse (%)	88%	55%	63%	0.07
Father of an infant (%)	36%	9%	35%	0.19
Number of children^d^	1.4±0.6	1.5±0.9	1.7±0.8	0.43
Hours of childcare per day^c^	2.8±4.0	3.0±3.1	4.0±4.5	0.18
Nanny (yaya) childcare help (%)	0%	0%	3%	1.00
Grandmother childcare help (%)	18%	9%	10%	0.53
**Health-related variables**				
Fat-free mass (kg)	47.8±5.5	47.5±3.9	48.1±6.8	0.94
Triceps skinfold (mm)	15.0±7.4	15.5±6.2	15.2±7.2	0.98
Sick since last survey^e^ (%)	65%	73%	84%	0.07
Self-reported stress, PSS	17.2±3.8	19.0±4.0	17.1±3.6	0.22
Nighttime sleep duration (hours)	8.4±1.6	8.2±1.9	8.3±1.4	0.94
Sleep quality^f^	3.5±1.8	3.0±2.2	3.8±2.2	0.39

a results of ANOVA or Fisher's exact test unless indicated otherwise. All variables reflect 2009 data.

b highest grade completed.

c results of negative binomial regression.

d results of Poisson regression.

e since 2007.

f number of days per week that subject reported feeling rested at waking.

## Results

Table 1 provides descriptive statistics for the study subjects, grouped according to their familial sleeping arrangements. Fathers who practiced same surface cosleeping showed a borderline trend towards living in households with more people compared to fathers who slept solitarily (p<0.1), although the groups did not significantly differ in terms of socio-economic status (educational attainment; p>0.7). Given that cosleeping appears normative in this sample (Table 1), we also tested whether men who slept separately from their children differed in crucial characteristics that might confound relationships with T. Same surface cosleepers did not differ from other fathers in having young children or being involved in day-to-day childcare (both p>0.15). Families also received little childcare assistance from either nannies or grandmothers regardless of their sleeping arrangement (both p>0.5). Across the 3 categories, there were no significant differences in terms of fat-free musculature or adiposity (both p>0.9). There was a borderline trend for solitary sleeping fathers to have sex more frequently (p<0.1). There were no observed differences between the 3 groups of fathers for variables that might affect health, as self-reported psychosocial stress, nighttime sleep duration, and self-reported sleep quality were comparable (all p>0.2). However, same surface cosleeping fathers (84%) reported being ill since the last CLHNS survey (2007) at a higher rate than either roomsharing (73%) or solitary sleeping fathers (65%), which approached significance (p<0.1). Same surface cosleepers (7%) were not more likely to report being hospitalized with a serious illness compared to solitary (6%) or roomsharing fathers (0%) (p = 1.0).

**Figure 1:**
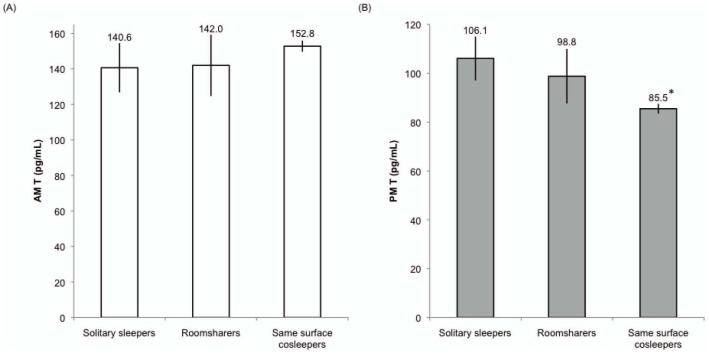
A–B: Values of follow-up (2009) AM T (1A) and PM T (1B). Values are adjusted for time of saliva collection and are derived from regressing T on familial sleeping arrangements, controlling for covariates, with solitary sleeping fathers as the comparison group (see Table 2). * p<0.05. Error bars indicate s.e.m.

**Figure 2:**
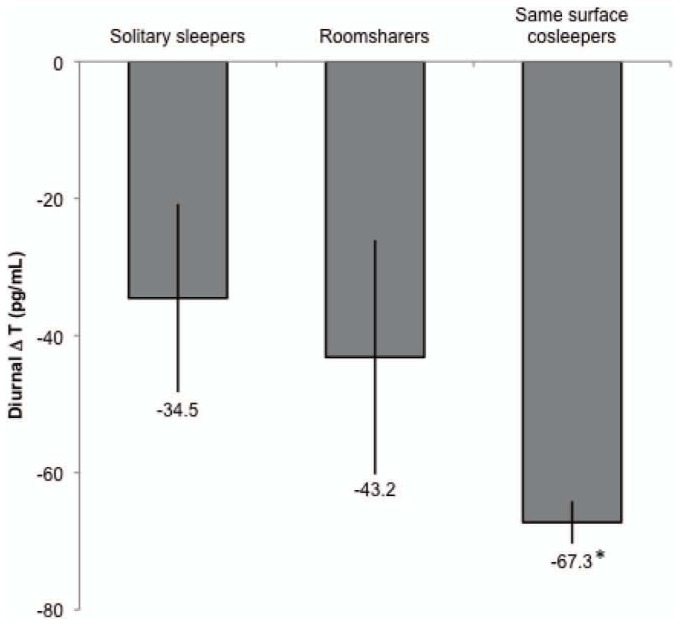
Diurnal change (Δ) in T at follow-up (2009). Values are derived from regressing ΔT on familial sleeping arrangements, adjusting for covariates, with solitary sleeping fathers as the comparison group (see Table 3). * p<0.05. Error bars indicate s.e.m.

Because we hypothesized that fathers' nighttime proximity to their children might affect their T, we predicted follow-up (2009) AM and PM T from fathers' familial sleeping arrangements (Table 2; Fig. 1A–B). While there were no significant differences for AM T across the 3 sleep conditions, men who slept on the same surface as their children had significantly lower PM T compared to men who slept solitarily, which remained significant after controlling for covariates (p<0.05). We also tested whether men's diurnal change in T was predicted by familial sleeping arrangements, finding that same surface sleepers had a significantly greater decline in T from morning to evening compared to solitary sleeping fathers (p<0.05; Table 3, Fig. 2). This also remained significant after controlling for covariates. Adjusting the models for self-reported illness between 2007 and 2009 did not affect the results (not shown).

**Table 2:** Predicting follow-up (2009) testosterone (T) from paternal sleep location^a^.

	*AM T*				*PM T*			
	Model 1	p	Model 2	p	Model 1	p	Model 2	p
**Sleep location** b								
Roomsharers	3.79±22.1	0.9	1.34±22.1	1.0	−6.60±14.2	0.6	−7.29±14.3	0.6
Bedsharers	14.70±14.2	0.3	12.14±14.2	0.4	**−19.72±9.1**	**0.03**	**−20.58±9.2**	**0.03**
**Fatherhood variables**								
Father of infant^c^			−5.09±6.3	0.4			−1.40±4.1	0.7
Number of children			7.33±3.7	0.05			1.80±2.4	0.4
Hours of care per day			0.62±0.7	0.4			0.35±0.4	0.4
*Model R^2^*	0.004		0.019		0.016		0.020	

a values are β ± SE of T adjusted for sampling time.

b excluded comparison group: fathers who slept separately from their children.

c excluded comparison group: fathers without an infant.

**Table 3:** Predicting diurnal change (Δ) in 2009 testosterone (T) from paternal sleep location^a^.

	*Diurnal* Δ*T*			
	Model 1	p	Model 2	p
**Sleep location** b				
Roomsharers	−10.39±21.8	0.6	−8.62±21.9	0.7
Bedsharers	**−34.41±14.0**	**0.02**	**−32.72±14.1**	**0.02**
**Fatherhood variables**				
Father of infant^c^			3.68±6.2	0.6
Number of children			−5.53±3.6	0.1
Hours of care per day			−0.27**±**0.7	0.7
*Model R^2^*	0.021		0.029	

a values are β ± SE of ΔT adjusted for sampling time.

b excluded comparison group: fathers who slept separately from their children.

c excluded comparison group: fathers without an infant.

**Figure 3:**
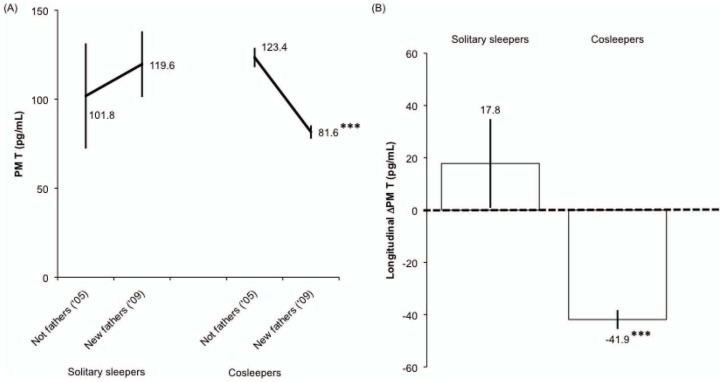
A–B: Changes in PM T between baseline (2005) and follow-up (2009). Analyses were restricted to men who transitioned from being non-fathers in 2005 to parents in 2009. Values are adjusted for time of saliva collection, stratified according to whether fathers were solitary sleepers (n = 9) or cosleepers (n = 200). Statistical comparisons reflect that cosleeping fathers had a significantly greater decline in PM T compared to solitary sleeping fathers. *** p = 0.001. Error bars indicate s.e.m.

To clarify whether men with lower T were more likely to cosleep with their children, we next tested whether baseline (2005) T predicted familial sleep practices at follow-up (2009) among men who were non-fathers at baseline (n = 209). Because there were only five men in the “roomsharing” category for this analysis, they were placed in the same category as same surface cosleepers. Neither baseline (2005) AM T (OR 1.00, 95% CI 0.99–1.01; p>0.9) nor PM T (OR 1.01, 95% CI 0.99–1.03; p>0.2) predicted whether men slept near their children or slept solitarily at follow-up (2009). In addition, using this same sub-sample of men, we tested whether cosleeping predicted a greater longitudinal change in T between baseline and follow-up. In these models, there were no differences between solitary and cosleeping fathers for long-term change in AM T (β −4.51, SE ± 30.71, R-squared 0.0001; p>0.8), with both groups showing declines over the follow-up period. However, men who reported cosleeping at follow-up had a significantly greater decline in PM T (β −59.71, SE ± 17.38, R-squared 0.054; p = 0.001; Fig. 3A–B) over the 4.5-year follow-up period compared to solitary sleeping fathers, whose PM T increased, on average. All results were comparable when roomsharing and same surface cosleeping men were treated as separate categories.

**Figure 4:**
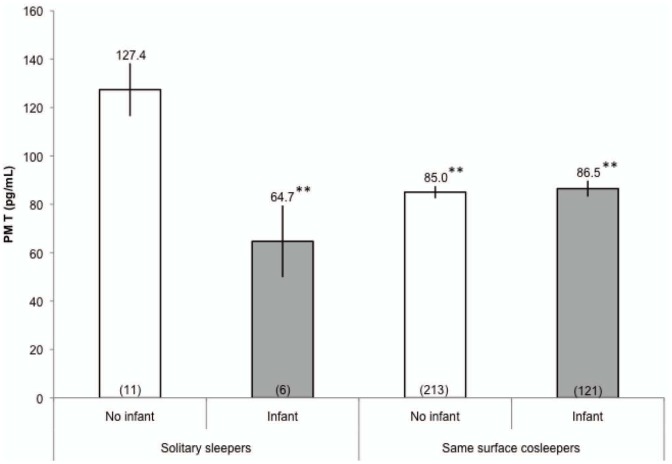
Follow-up (2009) PM T, stratified by sleeping arrangements and whether men were fathers of infants. White bars indicate fathers without infant-age children. Gray bars indicate fathers with infants. Sample sizes listed in parentheses. Statistical comparisons reflect one-way ANOVA of hormonal values, with Bonferroni multiple comparison tests. Values are adjusted for time of saliva collection. Comparison group: solitary sleeping fathers without infants. ** p<0.01, * p<0.05. Error bars indicate s.e.m.

Because it was previously found that fathers with younger children had lower T in this cohort, we used ANOVA to test whether follow-up T differed among fathers with and without infant-aged children, stratified by sleeping arrangement (Fig. 4). There were no significant group differences for AM T (all p>0.4). Solitary sleeping fathers with infants had significantly lower PM T than solitary sleeping fathers without infants (p<0.01). Same surface cosleeping fathers with infants (p<0.01) and without infants (p<0.01) also had significantly lower PM T than solitary sleeping fathers without an infant-aged child (Fig. 4). Other comparisons for PM T did not reach significance (all p>0.8). Among roomsharing fathers, only 1 man had an infant, and thus roomsharing fathers were excluded from this particular analysis (see Fig. 4).

## Discussion

A growing body of evidence indicates that in many cultural settings fathers have lower T than non-fathers [34], [35], including recent evidence that first-time fatherhood causes men's T to decline over a multi-year period [38]. There are also cross-cultural data showing that fathers' involvement in the daytime care of their children may be a principal determining factor in whether and the degree to which fathers have lower T than other men [13], [38]. However, nothing is known about the role that nighttime father-child proximity may play in influencing paternal T. Thus, we used data from a large sample of 25–26 year old fathers in Metro Cebu, Philippines to test whether fathers' T differed if they coslept with their children at night or slept separately from them. A very high percentage (92%) of fathers in this sample reported practicing same surface cosleeping. We found that fathers who slept on the same surface as their children had significantly lower PM T and a significantly greater diurnal decline in T from waking (AM) to bedtime (PM) compared to men who slept solitarily. Drawing on men who became first-time fathers during the 4.5-year study period, we also showed that men's baseline T did not predict whether they practiced solitary sleeping or cosleeping at follow-up. However, among these same men, those that coslept with their children had a significantly greater longitudinal (over 4.5 years) decline in PM T compared to new fathers who slept separate from their children. These results are the first to show that T is comparatively lower among fathers practicing same surface cosleeping compared to solitary sleeping fathers and suggest that cosleeping may cause T to decline and/or remain relatively low.

In combination with limited ethnographic observations [44], [56], [65], a recent cross-cultural internet-based survey that sampled parents in the Philippines [57], and a previously published report that 99% of infants slept with their mothers in Cebu [58], our results indicate that familial cosleeping is culturally normative in the Philippines. Although in many popular and scholarly publications on familial sleeping practices same surface cosleeping is reduced simply to the label “bedsharing” [57], [66], [67] the practice of parents and children sleeping together takes hundreds of diverse forms across cultures, most of which are not ordinarily characterized by the use of a “bed,” in the sense of the elevated mattress and box spring commonly used in the United States and much of Europe, Australia, and Canada. In this study, our respondents replied to a question using the Cebuano term “*higdaanan*,” which literally translates as “something you sleep on, whether a bed, a mat, or a mattress on the floor.” Thus, though we cannot distinguish the specific forms of same surface cosleeping in this sample, it most likely involved fathers sleeping with their partners and child(ren) on various kinds of mats, thin mattresses, or blankets on the floor of their homes.

In American and many European societies, same surface cosleeping is a controversial practice. Certain medical and public health organizations argue that it poses a risk to child health under all circumstances [68]–[71]. Elsewhere it has been levied that familial cosleeping may also negatively affect marital quality and parental sleep [72], though evidence from scientific studies to-date suggest that families that routinely sleep with their children generally avoid such relationship discord and sleep quality problems [43], [73], [74]. In the health-related parameters we assessed, we found no significant differences between fathers based on their familial sleep practices for self-reported psychosocial stress, sleep duration, or sleep quality, all of which could potentially affect T, but we did observe a trend indicating that same surface cosleepers had a tendency to have been ill at a higher rate between 2007–2009. We also found a statistical trend for cosleeping fathers to be less likely to have sex with their partners on a weekly basis compared to solitary sleeping fathers. The extent to which this might be disruptive to marital cohesion in this cultural setting is presently unclear but warrants exploration in future studies.

Previously, in a separate study of men from this same sample, it was shown that men's T as single non-fathers did not predict their caregiving levels 4.5 years later at follow-up and that fathers who were the most involved with childcare at follow-up had the lowest T [38]. Here we document similar findings in relationship to familial sleeping practices. Among men who transitioned from being non-fathers at baseline to being new fathers at follow-up, baseline T did not predict whether they coslept or slept separately from their children at follow-up. We also found that cosleeping fathers had a significantly greater longitudinal decline in PM T compared to solitary sleeping fathers, whose PM T increased, on average, between baseline and follow-up. Together these findings are suggestive that the lower evening T among same surface cosleeping fathers resulted from these fathers sleeping in close proximity to their children at night. Limited results from other species show that high T interferes with paternal investment, leading to lower offspring growth and reduced survival [75], [76], and preliminary evidence from studies of human males suggests that lower T men are more sensitive to child needs [27], [30]. Thus, it is possible that decreases in paternal T associated with cosleeping could have beneficial implications for children. There is evidence from industrialized societies in many parts of the world that children of highly invested fathers fare better in many developmental domains, including, for example, greater self-esteem and socialization skills, higher academic performance, and lower delinquency [77], [78]. Thus, though it remains speculative at this juncture, lower T could amplify the beneficial effects of daytime paternal care and nighttime cosleeping, facilitating and/or enhancing fathers' responses to their children in those contexts, thereby contributing to better child health and development outcomes.

It is also plausible that same surface cosleeping fathers have lower T as a result of sleep disruption that is not experienced by solitary sleeping fathers. Notably, we found no differences in self-reported sleep duration or sleep quality based on familial sleeping arrangement in our study, suggesting that these factors are unlikely to account for the documented differences in T, although issues of self-report reliability in these domains have been raised [79]. In addition, though no polysomnographic studies have been done on cosleeping fathers, prior research comparing polysomnography data from routinely bedsharing and solitary sleeping mothers revealed that bedsharing mothers had more transient, microarousals than mothers sleeping alone, but the two groups did not differ in time awake after sleep onset [43]. While it remains to be seen whether similar polysomnographic-observed arousal patterns translate to fathers, laboratory studies have shown that extreme methods of sleep fragmentation, i.e. waking men up every 20 minutes throughout the night, lead to reduced T production [80]. Further research is needed, generally, to assess causal relationships between naturalistic arousal patterns and men's T and, specifically, in the context of familial sleeping arrangements.

Fathers who practiced same surface cosleeping also showed a significantly greater diurnal decline in T from waking (AM) to evening (PM) compared to the decline seen in solitary sleeping fathers. It is noteworthy that while AM T did not differ by sleeping arrangement both the diurnal decline and PM T did. Recent studies have shown that short-term (∼20–30 min) periods of father-child interaction have almost no immediate effect on paternal T [30], [54], [55]. Thus, it is possible that the effects of father-child interaction in reducing fathers' T may take hours to come to fruition, which could help explain why same surface cosleeping fathers show a steeper diurnal decline in T and lower PM T overall, before going to bed, rather than an immediate effect on their AM T.

The physiological pathways through which this delayed effect might be possible are not well understood. It seems likely the process would be mitigated via the hypothalamic-pituitary-gonadal (HPG) axis, rather than through alternative physiological pathways that might rapidly affect circulating unbound T [81]–[84]. If father-child sleep proximity causes changes in neurobiological function that reduce hypothalamic production of gonadotropin-releasing hormone (GnRH) and/or pituitary production of luteinizing hormone (LH), perhaps through the downstream effects of neurotransmitters/neurohormones such as dopamine, serotonin, norepinephrine, and/or endogenous opiates, reduced T might not be observed until later sampling, such as our PM saliva collection. This would be generally consistent with the previous proposal that AM T levels reflect circadian-sleep biology and are more impervious to social stimuli in humans and other hominoids whereas PM T is more responsive to social and behavioral context [9]. That said, there is generally thought to be a 40–50 minute delay between changes in LH production and output of T from the testicular leydig cells [85], [86], so the neuroendocrine mechanisms by which close nighttime father-child sleep proximity might cause sustained lower production of T over the course of the day remain to be elucidated. Ideally, future studies will be able to integrate both nighttime laboratory-based hormonal analysis and daytime sampling in order to track men's T (and other biomarker) changes overnight as they sleep near their children (or not) as well as how their hormones then shift over the course of the day after waking.

We also found preliminary evidence that the effects of having young, infant-aged (1 year old or less) offspring may affect men differently based on their familial sleeping arrangements. Men who practiced same surface cosleeping had lower T regardless of whether their youngest child was an infant or older than a year when compared to solitary sleeping fathers whose youngest child was older than 1 year. Solitary sleeping fathers of infants also had lower T than their solitary sleeping counterparts without infant-aged children, though there were few men in this category (n = 6). Although these analyses are somewhat limited by small sample sizes in the solitary sleeping categories, our findings tentatively suggest that same surface cosleeping fathers may maintain lower T regardless of whether they have especially young children whereas solitary sleeping fathers' T may increase once offspring move out of infancy and become toddlers and beyond. These possibilities merit exploration in future longitudinal research.

This analysis has limitations that warrant mentioning. First, we asked fathers only about their sleeping arrangements on the night before salivary sampling, not their habitual activities. To our knowledge, families practicing cosleeping in Cebu generally do so regularly and for the entirety of the night, at least in part because of household space constraints. This differs from a common practice in more affluent societies in which infants and young children have their own bedrooms and are brought to the parents' room for portions of the night before being returned to their own sleeping quarters [87]–[89]. Thus, though presently there are no relevant scientific data from the Philippines available on the subject, it seems likely that men's self-reports of their sleeping arrangements from the prior night are generally reflective of their more routine sleeping arrangements. However, there is also a chance that fathers who normally slept separately from their children might have been near them the night prior to sampling because of some extenuating circumstances, e.g. a distressed child or maternal absence. Thus, it is possible that the “solitary sleeping” category is mildly underrepresented, though we think it unlikely.

Second, as we noted in the Methods, we collected the PM sample the night before the collection of the AM sample in order to reduce participant burden and minimize interviewer field logistics. This study design allowed us to schedule single interviewer follow-up visits to each participant to collect both tubes. Prior research suggests that salivary T measures represent relatively stable baselines for subjects [90]–[92], which we take to be true here, particularly given our study's sample size. We also relate the PM and AM T values to a behavioral measure (whether men cosleep with their offspring) that is believed to be habitual in this cultural context. Consequently, we think that this approach allows us to capture relatively stable between-individual hormonal variability based on familial sleeping patterns. However, we also realize this design differs from studies that conduct AM and PM sampling on the same day. Our approach renders the interpretation of the diurnal change in T potentially more difficult, as our calculation is not change over a single day (see Methods), diverging from other studies [e.g. 64]. However, if our T measurements are representative of relatively stable, day-to-day hormone levels, this issue is largely ameliorated. In total, we think it unlikely that an alternative research design, with both samples collected on the same day, would have substantially altered the results of the study or our interpretations thereof.

Finally, we collected single measurements of T at waking and in the evening for both the baseline and follow-up surveys [92]. However, our single measurements of saliva do not introduce bias, but merely reduce the reliability of our biomarker measures and thereby limit our ability to detect relationships between hormones as well as with other variables. The relatively modest R-squared values of our regression models would also be expected to increase with greater measurement reliability. That said, the effect sizes (β coefficients) for our significant regression models are sufficiently large to suggest that our results are biologically meaningful [6]. For example, in our study, bedsharing fathers had 19% lower PM T, on average, compared to solitary sleepers (Cohen's *d* = 0.55, based on group means and pooled SD), which is similar to the percentage differences in T observed among men before and after engaging in competition [93] or being exposed to visual sexual stimuli [94], [95] and is comparable in magnitude to the differences in T between men who engage in high versus low risk taking [24]. The impact of low measurement reliability in our study was partially compensated for by the fact that we collected saliva samples at standardized times in a sample of men that exceeds the size of most prior studies of human male socioendocrinology.

In summary, our study is the first to test for relationships between cosleeping and paternal physiology, showing that fathers who slept near their children on the same surface had lower evening T and greater diurnal declines in T compared to fathers who slept separately from their children. In addition, we showed that fathers' T might respond differently as children age based on how families sleep, as same surface cosleeping fathers maintained lower T regardless of whether they were fathers to infants whereas solitary sleeping fathers with older children had higher T. These results are generally consistent with the idea that human paternal physiology has an evolved capacity to respond to childcare and direct contact with children [10], [12], [38]. Future studies are needed to clarify whether differential effects of cosleeping versus solitary sleeping on men's T might influence their effectiveness as caregivers and potentially affect their children's development.
